# Psychological Impact of Congenital Chest Wall Deformities Among Adolescents and Young Adults

**DOI:** 10.3390/children13020237

**Published:** 2026-02-07

**Authors:** Elizabeth A. Barmash, Babetta B. Mathai, Sara A. Mansfield, Kyle J. Van Arendonk

**Affiliations:** 1 Center for Surgical Outcomes Research, Abigail Wexner Research Institute, Nationwide Children’s Hospital, Columbus, OH 43205, USA; elizabeth.barmash1@nationwidechildrens.org; 2 Division of Pediatric Surgery, Department of Surgery, Nationwide Children’s Hospital, Ohio State University, Columbus, OH 43210, USA; sara.mansfield@nationwidechildrens.org; 3 Department of Pediatric Psychology, Nationwide Children’s Hospital, Columbus, OH 43205, USA; babetta.mathai@nationwidechildrens.org

**Keywords:** chest wall deformity, pectus excavatum, pectus carinatum, psychosocial impact, quality-of-life

## Abstract

**Highlights:**

**What are the main findings?**

- Psychosocial burden, including impaired body image, social anxiety, and low quality of life, is significant in adolescents and young adults with pectus excavatum and pectus carinatum, often manifesting as social avoidance, concealment behaviors, and emotional distress.
- Surgical and non-surgical correction of chest wall deformities markedly improves body image, self-esteem, social participation, and mental health scores, with benefits sustained long-term.

**What are the implications of the main findings?**

- Routine psychosocial screening and incorporation of psychologists into chest wall deformity clinics are essential for optimizing outcomes.
- Expansion of insurance coverage beyond anatomic criteria to include psychological indications can help mitigate long-term emotional distress.

**Abstract:**

Progression of pectus excavatum and carinatum coincides with adolescence, a critical period for identity and self-esteem development. While clinical decision-making and insurance coverage have historically emphasized anatomic severity and cardiopulmonary functioning, increasing evidence suggests that psychosocial burden and quality of life (QoL) impairment represent central components of disease impact. A narrative review was conducted using the PubMed database to synthesize the current literature on the psychological impact of these deformities in adolescents and young adults, including body image distress, social functioning, and mental health effects before and after surgical and non-surgical correction, focusing on validated tools and qualitative studies. Across multiple cohorts, adolescents and young adults with chest wall deformities consistently report impaired body image, reduced self-esteem, social avoidance, and diminished QoL, even in the absence of diagnosable psychiatric disorders. Surgical and non-surgical corrections have positive effects in these domains. Psychological burden, therefore, represents a clinically meaningful component of chest wall deformities and should be considered alongside anatomic and physiologic criteria. Current evidence advocates for the integration of standardized psychosocial screening and support into evaluation and follow-up, which is essential for providing comprehensive, patient-centered care. Greater recognition of psychosocial outcomes may inform advocacy for broader treatment criteria, increasing accessibility among affected individuals.

## 1. Introduction

Congenital chest wall deformities encompass a broad spectrum of disorders, including thoracic ectopia cordis, sternal clefts, Poland syndrome, Jeune syndrome, and the two most common deformities—pectus excavatum (PE) and pectus carinatum (PC) [1]. The pathophysiology of chest wall deformities is poorly established, with proposed mechanisms related to impairments in the costal hyaline cartilage structure and function, and progression is commonly seen during periods of rapid vertical growth, such as puberty [1,2,3]. Patients often present with a range of physical symptoms during this time, including dyspnea, exercise intolerance, and chest pain, or with psychological distress related to body-image concerns [1,4,5]. Current insurance-approved indications for repair in the United States focus on these physical symptoms and the severity of the chest wall deformity, typically measured by the Haller Index (HI), which is calculated as the ratio of the transverse chest diameter to the anterior–posterior distance between the sternum and spine. However, there is a growing body of literature to support the impact of chest wall deformities on patients’ body image, mental health, and quality of life (QoL).

The progression of PE and PC coincides with adolescence, a critical period for identity and self-esteem development. Feelings of dissatisfaction with one’s appearance often emerge during this time as comparison with peers violates their desire to fit in and appear “normal”. As a result, body image scores in adolescents with chest wall deformities have been shown to be consistently reduced compared to healthy controls and are correlated with impaired QoL [6,7]. Distress related to self-image manifests in social avoidance, concealment behaviors, and social anxiety disorders [7,8,9]. Given the significant psychosocial impact of chest wall deformities, the treatment of PE and PC (surgical repair or bracing) has a positive effect on body image, self-confidence, and overall QoL [6,10,11,12,13,14,15,16,17,18,19,20,21,22,23,24,25,26,27,28].

This narrative review synthesizes the current literature on the psychological impact of PE and PC in adolescent and young adult patients, the validated assessments available to understand both patient and parental concerns, and the impact of chest wall correction on patient and parental well-being.

## 2. Methods

A narrative review was conducted to identify relevant studies published between January 1990 and November 2025 using the PubMed database. Search terms included the following combinations: “pectus excavatum”, “pectus carinatum”, “chest wall deformity”, “quality of life”, “psychological”, “psychosocial”, “mental health”, “body image”, “adolescent”, and “pediatric”. Additional articles were identified by a manual search of the cited references, including those referenced in systematic reviews. A reference librarian was consulted to supplement the search, and 21 relevant articles were identified, with 7 of them being new. Titles and abstracts were screened for relevance to the scope of this review. The selected studies are comprised of observational studies, systematic reviews, and validated instrument development studies that evaluate psychosocial outcomes in patients with PE or PC with an average age ≤ 25 years. Case reports, purely technical surgical descriptions, and studies without psychosocial endpoints were excluded. Abstracts without available full-text manuscripts and non-English language availability were also excluded.

## 3. Anatomic Basis of Chest Wall Deformities

PE is characterized by depression of the anterior chest wall and is the most common chest wall deformity, accounting for approximately 65–95% of all cases [1,5]. Although incidence and prevalence rates vary due to differences in diagnostic methods, it is estimated to occur in up to 8 per 1000 live births. Historically, PE was thought to have a male predominance as seen in clinical studies; however, a recent radiographic study demonstrated a female predominance [5,29]. While the exact mechanism of PE is not completely understood, there is consensus that the defect is present at birth but may not manifest until years later during the prepubertal period of rapid skeletal growth [1,3]. Proposed mechanisms relate to abnormalities in the costal hyaline cartilage structure and function, resulting in weakness or overgrowth and subsequent chest wall collapse. PE can occur independently or in association with other conditions, including scoliosis, Marfan syndrome, Ehlers–Danlos syndrome, Noonan syndrome, and neurofibromatosis [1,2,3,5]. Symptoms range from shortness of breath, exercise intolerance, lack of endurance, and chest pain to body image concerns [1,4,5]. Surgical management by the Nuss procedure, or less frequently the Ravitch procedure, is offered for patients with a Haller Index ≥ 3.25 in the U.S. [30]. Alternative therapies include an emerging external traction system to elevate the chest, called the taulinoplasty or Pectus Up procedure, and pectus implants, which correct the appearance but not the sternal depression of the chest wall, with a subpectoral silicone implant [31,32].

PC is the second most common chest wall deformity, characterized by protrusion of the anterior chest wall. Similar to PE, incidence and prevalence rates are difficult to establish, but PC is estimated to occur in 1 per 2500 live births, with a male predominance [33]. Symptoms are generally milder than in PE, oftentimes limited to psychological distress but sometimes also including thoracic pain, decreased endurance, or exertional dyspnea related to the fixed anterior–posterior diameter of the chest wall [1,5,34]. Orthotic bracing to apply compressive anterior-to-posterior force to depress the sternum is the first-line therapy, with reported success rates of 65–85% [1,5]. Surgical correction via the modified Ravitch procedure or a minimally invasive repair of pectus carinatum (known as an Abramson procedure or reverse Nuss procedure) is reserved for patients who fail conservative management [25,33,35,36].

## 4. Psychosocial and Mental Health Impacts

### 4.1. Body Image and Self-Esteem

Adolescence is characterized by a time of great physical, psychological, and social change during which individuals are developing their self-concept [10]. Body image is an important component, as dissatisfaction with one’s appearance is correlated with lower self-esteem and drives the development of emotional or psychological difficulties, including depression, anxiety, and social phobias [10,37,38]. Feelings of body distress are amplified by a normative part of identity development called adolescent egocentrism, which is a cognitive distortion that others are as preoccupied with their appearance as they are, due to an inability to discriminate between the thoughts of others and one’s own mental preoccupations [39]. As a result, adolescents are increasingly self-conscious and concerned about others’ opinions about their appearance [18,39]. As the timing of the typical progression of chest wall deformities aligns with this developmental stage, psychological distress related to bodily changes is amplified.

Reports on body image measures are significantly and consistently reduced in patients with both PE and PC [6,7,10,21], including when compared to healthy controls [40]. Through the use of standardized preoperative interviews, one study found that 40% of PE patients were constantly preoccupied with their chest, 62.5% felt shame, and 25% described a direct impact on self-image [6]. Another study comparing mild, moderate, and severe PE deformities based on HI found that 27–42% of individuals in the mild-to-moderate range and 39–55% in the severe range experienced negative sentiments related to their body image and self-esteem, with these sentiments strongly correlating with impaired QoL (Spearman correlation coefficient = 0.78). [7]. While much of the literature focuses on PE, patients with PC have also been shown to have a higher degree of body image and self-esteem impairment compared to healthy peer controls in smaller studies [40].

Findings regarding an association between body image-related distress and anatomic severity indices (e.g., Haller Index) vary. Interestingly, the majority of studies suggest a weak or no correlation [6,7,19]. One study did find a significant difference in psychosocial distress between those with the most (HI ≥ 6.0) and least (3.0 ≤ HI ≤ 3.9) severe deformities [9]. As many studies do not describe the range of severities included, it is difficult to discern if there is a true relationship between the severity of deformity and psychological distress. Moreso than the severity, perceived appearance and teasing by peers seem to strongly correlate with psychosocial distress [8,9].

### 4.2. Social and School Functioning

Impairments in body image and self-esteem extend into social and school functioning, with adolescents consistently demonstrating avoidance of social interactions [12,16,17,40,41]. One study on PE found that social functioning was impaired in 26% and school functioning in 42% of individuals [7]. Social anxiety is also common, with one study finding that 43% of patients with PE had scores indicative of possible social anxiety disorder [42]. Individuals also perceive that they are avoided by others due to their deformity and report concerns that their chest will be an obstacle in intimate relationships [6]. There are conflicting reports on whether PE versus PC has a larger impact on social functioning. One study found significantly worse distress and avoidance in new and unfamiliar situations for patients with PE compared to those with PC or the healthy control group, while another found that social activities were more frequently impaired in patients with PC compared to PE [8,40].

Concealment behaviors are commonly performed to accommodate the distress related to body image and social factors, with studies demonstrating 44 to 57% of adolescents intentionally hide the deformity from public view [6,9]. Some of these behaviors include layering clothing, folding their arms in front of their chest, and avoiding social situations that lead to chest exposure, such as swimming and athletics [6,9,10,12,18,21,41]. Qualitative studies describe related constant and pervasive psychological distress that is reinforced by the comments, thoughts, and actions of peers [10,12], with one patient stating, “Some people would…find out and be like poking at my shirt and stuff” [10].

Teasing plays a major role, with studies highlighting that an alarming percentage of individuals with PE and PC have experienced or worry about teasing by peers due to the appearance of their chest wall, and importantly, more than 60% of these individuals have reported suffering from these comments [6,9,12]. This actual and anticipatory teasing produces hypervigilance to keep the deformity a secret and increases the frequency of psychosocial problems [9,12], with one study noting a 2.9 times higher odds of psychosocial problems for those who have been teased compared to those who have not [12]. Given the impact of others’ opinions, it is not surprising that dissatisfaction with one’s chest appearance and prior teasing about the deformity are motivating factors for patients seeking treatment [9].

The psychology of adolescence may explain the strong impact that peer perceptions have on the distress experienced by individuals with chest wall deformities. One study exploring patient–parental concordance for body image and self-esteem found that patients experienced negative sentiments regarding body image to a greater extent than their parents perceived, while the QoL findings were the opposite, with parents overestimating the impairment experienced by their children [7]. This finding may be related to egocentrism and the amplified weight placed on physical appearance by adolescents. Another study compared physical appearance ratings between patients with PE and PC to adolescent and adult raters who did not have a chest wall deformity, and the findings support this differential perception that occurs with age. Adults rated patients more favorably than the self-ratings of the patients with PE and PC, indicating the patients’ greater self-perceived impairment in appearance [43]. When comparing adolescent ratings to the self-ratings of the patients with PE and PC, no difference was found, suggesting that adolescent peers were more closely aligned with how the patients saw themselves. This fluctuating value placed on body image over time may be explained by pubertal developments and the formation of identity and should be considered when caring for adolescent patients.

### 4.3. Mental Health

Children and adolescents with PE and PC consistently demonstrate mental health vulnerability pre-operatively, although these often manifest as subclinical symptoms rather than meeting formal diagnostic thresholds for psychiatric disorders. The most consistent findings include elevated levels of anxiety and social anxiety. Case–control data comparing PE, PC, and healthy controls show that patients with PE have significantly higher anxiety scores on the Brief Symptom Inventory, a screening psychological assessment, compared to PC and controls [8]. Social anxiety was identified in 42% of patients with PE in another study, with increasing symptom burden correlating with decreased QoL and increased body image concerns (Spearman correlation coefficient 0.72 and 0.64, respectively) [7].

Subclinical depressive symptoms may also be common among patients with PE, but these findings are more variable. In one study, 57.5% of participants screened positive for depression on the Self-Rating Depression Scale (SDS) [44] while another study found no difference compared to healthy controls [40]. When emotional difficulties are present, however, they can be quite severe. In a qualitative study exploring the lived experiences of individuals with PE, one participant described suicidal thoughts related to the problems caused by their chest, stating “like, it’s not worth living if you’re gonna hide yourself all the time” [41]. This comment again demonstrates the interplay between self-image and psychological symptoms, and as such, it makes sense that there appears to be a correlation between anxious and depressive symptoms in those with chest wall deformities. In one study, patients with PE had significantly higher scores on the Child Behavior Checklist (CBC) combined anxious/depressed subscale compared to controls, while another study identified that depressive symptoms, assessed via the Mood and Feelings Questionnaire (MFQ), correlated closely with social anxiety (Spearman correlation coefficient 0.70) [7,9].

Despite elevated symptom scores, there does not appear to be an association between chest wall disorders and clinical diagnoses of mental health disorders per formal diagnostic criteria. One study conducting structured clinical interviews found no increase in the prevalence of overall diagnosed mental disorders among PE or PC patients compared to controls [40]. However, negative body self-evaluation was identified as the strongest predictor of mental health-related QoL. This study also found that patients with PC had both a higher degree of body image impairment and lower mental component scores on screening assessments compared to those with PE and healthy controls, again emphasizing that body image impairment is a key determinant of psychological strain.

While these symptoms infrequently meet the diagnostic threshold for psychiatric disorders, they still significantly impact QoL, self-esteem, and social functioning. These findings highlight that subjective perception of appearance, rather than anatomic severity, drives psychological distress and internalize problems that lead to mental health disorders. These findings reinforce the need for routine psychosocial screening and early intervention in the care of patients with chest wall deformities.

## 5. Quality of Life (QoL) Assessment Tools

Assessing QoL in children with PE and PC is essential as psychosocial burden often exceeds physiologic impairment. While surgical criteria traditionally rely on anatomic severity (e.g., Haller Index), patient-reported outcomes provide a more nuanced understanding of functional limitations, emotional well-being, and social participation. Over the past two decades, several validated instruments, including generic and condition-specific questionnaires, have been employed to capture these dimensions.

### 5.1. Validated Questionnaires

Studies evaluating the psychosocial impact of PE and PC often rely on generic QoL and health-related quality of life (HRQoL) instruments, such as the Pediatric Quality of Life Inventory (PedsQL) or Short Form 36 (SF-36), to understand functional impacts (Table 1). These tools are valuable, as they allow for benchmarking against normative data and can track global health improvements post-intervention. However, they often fail to capture the appearance-related distress that has been shown to lead many adolescents to seek surgical intervention [6,22,40]. Condition-specific instruments like the Pectus Excavatum Evaluation Questionnaire (PEEQ) and Single Step Questionnaire (SSQ) provide critical insight into body image and perceived physical ability, domains that are strongly associated with psychosocial well-being (Table 1). Most commonly, studies employ a variety of assessment tools for a comprehensive understanding of the impact of chest wall deformities on QoL.

#### 5.1.1. Pectus Excavatum Specific Assessment Tools

The Pectus Excavatum Evaluation Questionnaire (PEEQ) was the original PE-specific questionnaire developed by Lawson et al. in 2003 [11]. The PEEQ focuses on the domains of psychosocial well-being and physical functioning related to the individual’s chest appearance and concavity. Responses are given on a four-point Likert scale to reflect either the happiness or frequency of each component. Many studies using the PEEQ have shown the Nuss procedure to have a positive impact [11,15,19,45,46,47]. The tool has also been applied to patients with PC, both with and without adaptations [48,49]. Since its development, the PEEQ and its adaptations have been widely used to understand the QoL implications of chest wall deformities and how QoL is influenced by disease management, thereby increasing visibility of the psychological impacts of PE and PC.

The Nuss Questionnaire modified for Adults (NQ-mA) was adapted from the PEEQ by Krasopoulos et al. for use in adults through minor wording changes [13]. The NQ-mA also reverses the scoring of the first three questions to allow for summation of scoring, with higher scores indicating a better QoL. The questionnaire has also been used in the PC population, with one study renaming the survey to NQ-mP while keeping the questions and scoring the same [50]. Similar to the PEEQ, many studies using the NQ-mA have demonstrated a positive impact of surgical intervention on QoL for both PE and PC [13,14,23,40,50,51,52].

The Single Step Questionnaire (SSQ) was developed by Krasopoulos et al. to provide a simpler assessment more relevant to young adults, covering both pre- and post-operative feelings and outcomes [13]. The assessment was derived from the PEEQ and consists of 16 questions, with its design intended to allow for the degree of satisfaction from surgical intervention to be assessed not only by a single question, but also through the overall score. The nine studies reviewed that have used the SSQ for adolescents and young adults have all shown high levels of satisfaction following surgery [13,14,20,21,23,24,48,53]. While the questionnaire was created and validated for use at a single post-operative timepoint, Zuidema et al. demonstrated improvements at four timepoints following the Nuss procedure, thus expanding its use [53].

#### 5.1.2. Pectus Carinatum Specific Assessment Tools

The Pectus Carinatum Body Image Quality of Life (PeCBI-QOL) Questionnaire was created by Paulson et al. to address the gap of limited PC-specific assessment tools [54]. Following a development phase and refinement process, the final questionnaire for patients includes 18 items focused on four domains: body image disturbance, treatment motivation/engagement, physical limitations, and social disadvantage. The parent component includes 15 items on the same domains, except with the addition of social disadvantage. The initial study on PeCBI-QOL found that following treatment, there was a significant improvement in overall PeCBI-QOL scores, with moderate patient–parent concordance [54].

#### 5.1.3. Generic Assessment Tools

Short Form 36 (SF-36) is a generic HRQoL instrument created for adults that consists of 36 questions across two broad health indicators, the Physical Component Score (PCS) and Mental Component Score (MCS), which are divided into eight sub-domains. Subscales and summary scores range from 0 to 100 points, and higher scores indicate better HRQoL [23,55]. The SF-36 has been applied to at least five studies examining chest wall deformities, and a shorter version consisting of 12 questions has also been utilized [23,40,42,48,56,57].

Child Health Questionnaire (CHQ) is a pediatric-focused generic HRQoL instrument that assesses physical, emotional, social, and family functioning through parent and self-reported formats [58]. There are a variety of survey lengths, including short forms CHQ-CF45 and CHQ-PF28, and full-length CHQ-CF87 and CHQ-PF28 for both children and parents. Responses are given on a four or five-point Likert scale, with higher scores indicating better HRQoL. Given its multi-dimensional focus for both children and parents, the questionnaire and adaptations have been used extensively in the chest wall deformity population [14,16,45,56,59].

The Symptom Checklist-90 (SCL-90) and its revised version (SCL-90-R) have been used to evaluate a broad range of psychological symptoms and mental health disorders. The tool assesses three global indices—Global Severity Index (GSI), Positive Symptom Distress Index (PSDI), and Positive Symptom Total (PST)—and nine primary symptom dimensions, including somatization, obsessive-compulsiveness, interpersonal sensitivity, depression, anxiety, hostility, phobic anxiety, paranoid ideation, and psychoticism. The tool has been used in at least three studies on PE [21,22,44].

The Pediatric Quality of Life Inventory (PedsQL) is a pediatric HRQoL instrument that has been widely used in pediatric research. PedsQL covers the domains of physical, emotional, social, and school functioning and has been applied in at least two studies of PE and PC [7,8].

Several studies have utilized semi-structured interviews to gain a broader understanding of how chest wall deformities have impacted patients in their own words [6,10,12,41]. Themes of social belonging, well-being, self-esteem, satisfaction, and empowerment have been identified, which correlate with the themes seen in commonly utilized questionnaires.

**Table 1:** Commonly utilized questionnaires in pediatric chest wall deformity research.

Instrument	Domains	Target Audience	Frequency of Use	Strengths	Limitations
Pectus-Specific Questionnaires					
Pectus Excavatum Evaluation Questionnaire (PEEQ) [11]	Psychosocial & Physical	Developed for PE; has parent form	8 studies	Condition-specific, sensitive to cosmetic outcomes, short (12 questions), parental version	Limited mental health assessment
Nuss Assessment Questionnaire Modified for Adults (NQ-mA) [13]	Psychosocial & Physical	Adapted PEEQ for adult PE use	7 studies	Allows for score summation	Limited mental health assessment
Single Step Questionnaire (SSQ) [13]	Physical, Psychosocial, Postoperative pain & satisfaction	Developed for PE	9 studies	Comprehensive assessment including surgery-related questions, short (16 questions)	Limited to post-operative use
Pectus Carinatum Body Image Quality of Life Questionnaire (PeCBI-QOL) [54]	Body Image, Treatment Motivation, Physical Limitations, & Social Limitations	Developed for PC	1 study	Condition-specific, sensitive to cosmetic outcomes, parental version	Limited mental health assessment
General Questionnaires					
Short-Form 36 (SF-36)	Physical, Social, Physical Role Limitations, Emotional Role Limitations, Mental Health, Vitality, Bodily Pain, & General Health	Adult validated; used in adolescents	5 studies	Norm-based scoring, 36 questions	Not pediatric-specific
Child Health Questionnaire (CHQ) [58]	Physical & Psychosocial	Parent (PF50) and child forms (CF87)	5 studies	Comprehensive domains, parental version	Lengthy, generic
Symptom Checklist 90 (SCL-90)	Psychological & Psychiatric Conditions	General	3 studies	Captures key mental health symptoms and disorders	Not HRQoL, narrow focus
Pediatric Quality of Life Inventory (PedsQL)	Physical, Emotional, Social, & School	Pediatric patients	2 studies	Broad pediatric HRQoL coverage	Lacks body image specificity

## 6. Surgical and Non-Surgical Treatment: Psychological Outcomes

Psychosocial outcomes are central to the management of PE and PC, as body image concerns and emotional distress often outweigh physiologic limitations [23,51]. Both surgical and non-surgical interventions aim to not only correct the anatomic chest wall deformity but also improve mental health, self-esteem, and QoL.

### 6.1. Surgical Management

#### 6.1.1. Motivations for Surgery

The motivation to undergo surgical intervention for chest wall deformities varies based on psychological burden and physical symptoms. Motivations for those with PC are predominantly related to physical appearance, with different studies finding that 64–100% of patients sought surgery solely for appearance-related reasons [23,40,50]. Although less commonly reported, physical functioning was still identified as a motivating factor when considering surgery [23,40]. Patients with PE are also concerned about appearance, with 11–100% of individuals, depending on the study, citing it as the primary motivator [6,24,40]. However, physical symptoms play a more substantial role in PE than PC, with 21–31% electing for surgery due to physical symptoms alone [6,24,40], and 51% for both appearance and physical symptoms [40]. Verbal reactions from others have also been reported as a meaningful driver for surgical intervention in 15% of patients in one study [6].

Patients who elect for surgery typically demonstrate more negative pre-operative body satisfaction scores in comparison to those who choose non-operative management, as found in one study comparing those with PE and PC by choice of management [18]. Many adolescents who desire surgery self-advocate for evaluation, with one study identifying 59% of patients who educated themselves regarding pectus-related clinics and surgery before approaching their parents [22]. Together, these findings underscore that surgery is often pursued in response to persistent appearance-related distress, with physical symptoms often contributing to a lesser degree.

#### 6.1.2. Postoperative Improvements

**Figure 1:**
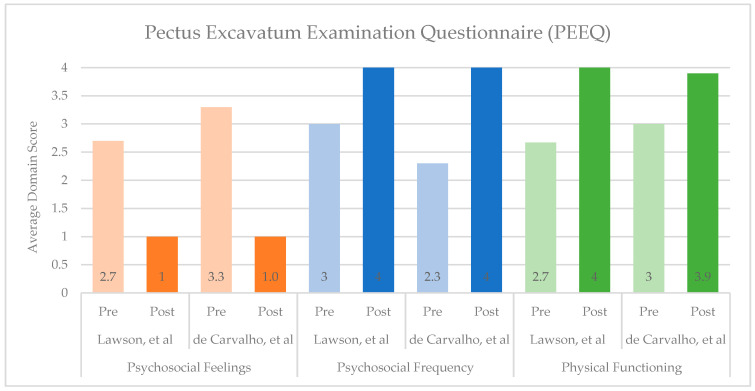
Summary of Pectus Excavatum Examination Questionnaire (PEEQ) scores before and after surgical correction of Pectus Excavatum or Pectus Carinatum [11,46]. Psychosocial Feelings include questions 1–3, answered on a 4-point Likert scale, where 1 = very happy and 4 = very unhappy (lower scores indicate more desirable experiences). Psychosocial Frequency includes questions 4–9, and Physical Functioning includes questions 10–12; both sets are answered on a 4-point Likert scale, where 1 = very often and 4 = never (higher scores indicate more desirable experiences).

**Figure 2:**
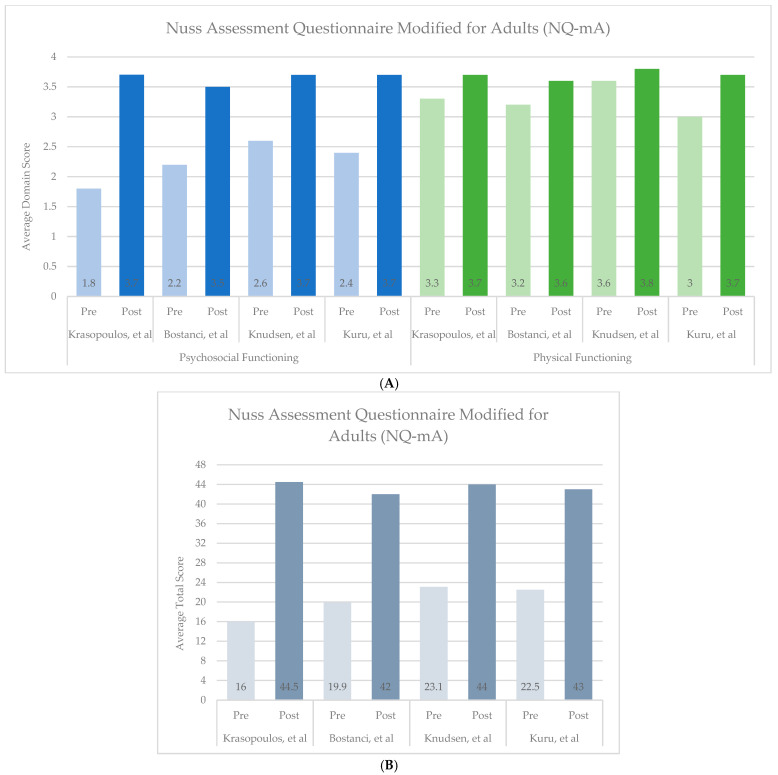
Summary of Nuss Assessment Questionnaire Modified for Adults (NQ-mA) scores before and after surgical correction of Pectus Excavatum or Pectus Carinatum [13,23,50,51]. (**A**). Psychosocial and Physical Functioning Domains. Psychosocial Function includes questions 1–9, answered on a 4-point Likert scale, where 1 = very happy and 4 = very unhappy for questions 1–3 and 1 = very often and 4 = never for questions 4–9. Physical Functioning includes questions 10–12 answered on a 4-point Likert scale, where 1 = very often and 4 = never. Higher scores indicate more desirable experiences. (**B**). Total Scores. Summation of scores across the psychosocial and physical domains. Answers can range from 12 to 48. Higher scores indicate more desirable experiences.

**Figure 3:**
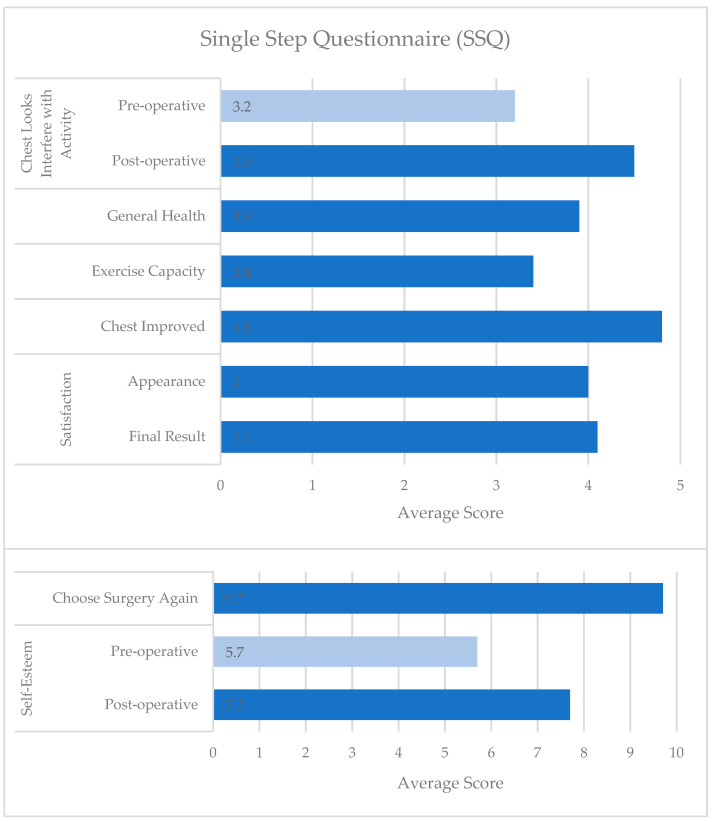
Single Step Questionnaire (SSQ) scores after surgical correction of Pectus Excavatum or Pectus Carinatum [13,20,23,24,53]. The survey is administered after treatment. However, two questions ask patients to recall their pre-operative self-assessment for comparison. Questions are scored on a 5-point Likert scale for chest appearance interfering with activity, general health, exercise capacity, chest improvement, and satisfaction. Self-esteem and the decision to have the operation again are scored on a scale of up to 10 points. Higher scores indicate more desirable experiences.

Surgical correction of PE and PC leads to significant improvements in both psychosocial and physical domains, as demonstrated by qualitative studies and quantitatively over time through condition-specific assessments. Of the eight studies reviewed that used the PEEQ, two reported pre- and post-operative scores, and both demonstrated improvements across all domains (Figure 1) [11,46]. Similarly, four of the seven studies that used the NQ-mA had pre- and post-operative scores available, with improvements seen in each domain and overall (Figure 2A,B) [13,23,50,51]. As the SSQ is typically administered at one point in time, sufficient data on individual questions were included in five of the nine studies reviewed, and the studies showed post-operative improvements and high satisfaction (Figure 3) [13,20,23,24,53].

**Body Image & Self-Esteem:**

Across multiple studies, surgical correction of PE and PC provides marked improvements in body image and self-confidence [11,12,13,14,15,16,17]. Post-operatively, questionnaire scores show that between 97 and 99% of patients with PE, depending on the study, were improved and happy with their chest appearance [19,20,24,46]. Furthermore, 90–100% felt comfortable having their shirt off and were satisfied with spending the rest of their life with their updated chest appearance based upon PEEQ and NQ-mA assessments [15,46]. PC patients show similar benefits, with one cohort demonstrating that their satisfaction with their appearance without a shirt on improved from 29% to 85% following the modified Ravitch procedure, and acceptance of their chest appearance for the rest of their life improved from 4% to 93% [23]. Semi-structured interview studies reinforce these findings, with adolescents and young adults reporting increased confidence in social settings, improvements in self-acceptance, and decreased worries about the perceptions of others [12,14,22].

Interestingly, one study found that following the Nuss procedure, PE patients had scored higher in nine CHQ scales compared to healthy control peers, indicating better HRQoL [14]. One possible explanation is that due to their significantly impacted self-esteem and preoccupation with their chest appearance at baseline, surgical correction of PE may lead to a more robust sense of confidence. This theory is supported by the findings of other studies, where the attribution of QoL to their chest wall, the ownership to seek care to improve their appearance, and post-operative feelings of normalcy allow those with chest wall deformities to feel an inflated sense of self-confidence [10]. As a result, patients with both PE and PC engage less in concealment behaviors, feel less bothered by their chest appearance, and become more willing to engage in social interactions after surgical correction [6,10,23].

The timing of these improvements has been cited to be most significant from the pre-operative state to the six-week timepoint after surgery, with score stabilization at six months and beyond [53,59].

**Social Functioning:**

Improvements in body image and self-esteem following surgical correction translate into improvements in social belonging and empowerment. Consistently, studies have found increased participation in sports and peer activities, decreased concealment behaviors, and improved comfort taking off their shirt in the locker room, all of which were previously avoided due to chest exposure [6,10,14,20,21,24]. These findings have been affirmed by systematic reviews [17,25,26]. In semi-structured interviews following the Nuss procedure, adolescents reported that the cessation of concealment behaviors led to feelings of relief, as they felt comfortable being open with peers about their PE and surgery [10]. PC patients show similar post-operative improvements, with NQ-mA scores demonstrating less reluctance to wear clothes that expose the chest in public, as well as decreased teasing and feelings of isolation [50].

**Mental Health:**

While the mental health disturbances seen in adolescents and young adults with PE and PC may not consistently meet formal diagnostic thresholds, significant post-operative improvements have been observed on a variety of questionnaires. Generic HRQoL questionnaires, such as the CHQ, have shown improved scores on the mental health scale [16] while significant reductions in the domains of depression and interpersonal sensitivity have been seen on the SCL-90 following PE repair [22,44]. Furthermore, both depressive symptoms and overall mental health problems in those with PE decreased from 57% to 29% and from 61% to 30%, respectively, from before to after surgery [44]. These improvements were independent of the Haller Index, reinforcing that psychosocial benefit is not severity dependent. Similar improvements have been observed for those with PC on both generic and condition-specific QoL assessments regarding feelings of sadness, depression, and general mental health [23,50].

**QoL & Satisfaction:**

Overall QoL improvement and satisfaction with surgical intervention have been strongly observed following correction of both PE and PC deformities [12,18,19,20,21,22,23,24]. Satisfaction is independent of the procedure performed (Nuss vs. Ravitch), and for patients with PE who underwent the Nuss procedure, high satisfaction has been reported at both six months post-operatively and after bar removal [19,20]. Across several studies, 85–96% of patients with PE reported that they would choose to have the operation performed again [19,20,24]. This overall high level of satisfaction has been confirmed on all five systematic reviews conducted on chest wall deformities and QoL [17,25,26,27,28].

Long-term studies have demonstrated that patients with PE maintain high satisfaction and long-lasting positive effects on their psychological state years following bar removal [20,22,47]. A minority experience persistent distress or require surgical revision, often related to asymmetry or recurrence.

#### 6.1.3. Parental Viewpoints

The distress felt by many adolescents does not go unnoticed by their parents, with a majority of studies identifying strong patient–parental score concordance [11,12,14,15,19,51,60]. Mirrored distress in parents is common, with 79% of parents expressing frequent concern about their child’s deformity pre-operatively, which drops to 16% post-operatively [19]. Parents also confirm marked improvements in their child’s psychosocial functioning, social self-consciousness, and overall confidence following surgical correction of both PE and PC [11,15,19,50,51]. Reduced concerns about the effects of PE on their child’s life, enhanced family dynamics, and fewer conflicts around concealment behaviors are common themes [11,14]. There appears to be no difference in parental perceptions based on surgical technique [19].

#### 6.1.4. Impact of Pain

Post-operative pain is common following both open and minimally invasive repair of chest wall deformities, peaking in the first four to six weeks and impairing initial return to normal activities, and then improving significantly by six months [12,20,24,53]. Long-term pain following bar removal is rare [20,24]. Some adolescents have expressed that the surgical pain is preferable to the ongoing emotional distress of living with the deformity, with one interviewee stating that “any amount of pain would probably be better than the emotional pain” [10,12]. Predictors of persistent pain include pain-specific psychological variables (pain anxiety, hypervigilance), highlighting the need for targeted interventions [61]. Overall, patients and families have reported that the risks and pain associated with surgery are well worth the benefits [12].

Advances in multimodal analgesia have dramatically improved early recovery following chest wall deformity repair. Early post-operative strategies included regional anesthesia with thoracic epidurals, opioids, and non-steroidal anti-inflammatory medications. More recently, intercostal nerve cryoablation has been introduced as an ideal solution to mitigate post-operative pain. Cryoablation has been shown to provide considerable improvements in post-operative opioid consumption and length-of-stay after the Nuss procedure [62,63]. While many patients with severe and symptomatic PE previously chose surgical intervention despite the expected post-operative course, the successful introduction of cryoablation may further encourage patients to consider surgical intervention when they otherwise would not have, given the dramatically improved post-operative recovery enabled by the use of cryoablation.

Finally, another approach being explored is providing patients and their parents with pre-operative psychological coping strategies for pain and surgical-related worries, which was found to be beneficial in a recent study [64].

### 6.2. Non-Surgical Management

Non-operative management of PC through orthotic bracing has shown similar psychosocial improvements to surgical intervention. Successful bracing is dependent on patient motivation and adherence, with high attrition rates observed when expectations are not met [48,49]. Adolescents who complete bracing report significant improvements in body image, self-esteem, mental health, social participation, and physical functioning on both generic HRQoL tools and condition-specific questionnaires such as the modified PEEQ and PeCBI-QOL [48,49,54,56]. These improvements were most significant in the first six months after starting bracing and demonstrated stability in the two years following [56]. Individuals who failed bracing or were non-compliant with bracing had minimal psychosocial and overall QoL benefit, resulting in continued distress and dissatisfaction [48,56]. However, both studies comparing successful and unsuccessful bracing found that participants were interested in trying bracing again if it meant that their outcome could be improved. Family involvement enhances adherence and satisfaction. Similar to surgical correction, there is considerable parent–child concordance in QoL improvements, and supportive families facilitate coping with the demands of prolonged bracing [48].

There are limited data available regarding the psychological changes following non-operative management of PE with the vacuum bell technique, making it challenging to draw any definitive conclusions regarding its impacts [21].

Intervention of any variety may not be mandatory for all patients, with recent consensus guidelines suggesting that many deformities are well-tolerated and that supportive care is sufficient [32]. Limited data are available on the long-term psychological outcomes of those who elect conservative management, as psychological burden is a common reason for presenting to surgical evaluation and differs from those who choose non-operative management [18]. No studies have determined a level of psychological harm that indicates an operation would or would not be beneficial.

## 7. Health Policy and Insurance Considerations

### 7.1. Haller Index and Functional Criteria

In the United States, insurance coverage for surgical correction of chest wall deformities is largely restricted to anatomic and physiologic criteria. For PE, most payers require a Haller Index ≥ 3.25 and objective evidence of cardiopulmonary compromise, such as abnormal pulmonary function tests (PFTs), echocardiography, evidence of cardiac compression, or exercise intolerance [65,66,67,68]. Coverage for PC is typically more restrictive, as bracing and surgical repair are often categorized as “cosmetic” in the absence of demonstrable cardiac or pulmonary compromise expected to improve post-repair [65,66,67,68]. These criteria may exclude patients whose primary concern is psychosocial functioning, despite evidence that such factors can meaningfully affect quality of life.

International practices vary, with several centers reporting performing corrective surgery for appearance-related indications alone, with favorable satisfaction and mental health outcomes [6,15,23,24,40,50]. Other regions have adopted more restrictive criteria. In the United Kingdom (UK), the National Health Service (NHS) decommissioned routine surgical correction of chest wall deformities in 2019 [69]. Eligibility in England is closely aligned with anatomic and physiologic thresholds, and a multi-disciplinary review makes the final determination. No coverage is provided for bracing or surgical correction for PC. Scotland permits broader access, with patients under the age of 16 able to access a multi-disciplinary team that promotes a non-operative approach first. Following the decommissioning of surgical correction, joint specialist societies issued best-practice guidelines recommending that psychological impact, such as low self-esteem, depression, and social withdrawal, be evaluated when determining eligibility for intervention [32,51].

The distinction between “cosmetic” and reconstructive surgery is important, and mislabeling corrective surgery as cosmetic can be misleading and potentially harmful. While cosmetic is traditionally defined as modification of *normal* anatomy for aesthetic preference, chest wall deformity repair addresses a congenital structural abnormality and aims to restore normal thoracic contour and function [70]. Reliance on the Haller Index as a sole metric can be problematic, as studies show inconsistent associations between HI and psychosocial distress or surgical complexity. Patients with indices < 3.25 have reported substantial QoL impairment with post-operative benefit, and surgeons internationally do not agree that HI should be an indication for repair [6,7,8,9,15,17,30]. Alternative metrics, such as the correction index (CI), which accounts for the depth of the deformity as a proportion in terms of the most prominent portion of the chest wall, have been proposed to offer better discrimination of severity [71]. Furthermore, several studies indicate that psychosocial impairment often exceeds physical limitations among individuals seeking repair [23,51].

### 7.2. Surgeon Advocacy for Broader Coverage

Surgeons and professional societies increasingly call for policy reform to address coverage gaps in psychosocial indications for repair. A recent international survey found that 84% of chest wall surgeons agree that body image disturbances should be an indication for corrective surgery, even in the absence of physiologic compromise [30]. There is limited consensus regarding routine imaging as part of the pre-operative evaluation. Aside from clinical photography, no specific test, including CT, MRI, echocardiography, ECG, or PFTs, reached broad agreement, with many surgeons reserving relevant cardiopulmonary studies for symptomatic patients. One center has opted against pre-operative CT due to the hazards of radiation exposure in young patients, given that Haller Index measurements do not reliably predict outcomes [24]. Surgeons appear to agree that functional indices should be used as an adjunctive measure, rather than as the sole measure to assess severity for repair [15,27,72]. Patients and surgeons have also advocated for increasing multidisciplinary support through the inclusion of psychologists in chest wall deformity clinics to routinely support the psychological needs of these patients [21,22].

The age at which repair should occur remains an important aspect that has not been fully elucidated. Early intervention may prevent years of emotional suffering and social isolation that have accumulated during adolescence [41,44]; however, current surgical timing considerations often focus on anatomic and technical aspects of the operation, and it is unknown if this aligns with optimal timing for psychological benefits. Survey data suggest that the youngest age for pectus repair should be 12 years old, though earlier intervention may be considered on a case-by-case basis [30].

## 8. Limitations and Future Directions

While narrative methods enable an integrative approach to summarize the literature, the conclusions of this review may be limited by the absence of systematic approaches that quantify the quality and rigor of the analyzed data. However, all five systematic reviews identified on the topic were included and had concordant findings. Additionally, the literature on this topic frequently utilizes small sample sizes and heterogeneous outcome measures through predominantly observational designs, and there is a paucity of longitudinal data. This limitation is even more apparent for PC, where all but one study included <50 patients. While parallel psychosocial impacts have been observed between PE and PC, the underrepresentation of data on PC may not be representative of the true burden. Studies infrequently capture the formal diagnosis of mental health disorders and instead focus on symptom burden, limiting the conclusions that can be drawn regarding the impact on mental health.

Research has consistently demonstrated the psychological and QoL improvements following chest wall correction, yet insurance criteria have limited the number of individuals eligible for repair. Ongoing research should include individuals who do not meet the Haller Index threshold of ≥3.25 to better establish mental health criteria for repair. This research can support advocacy for expanding insurance coverage criteria to include psychosocial impairments and aligning reimbursement with evidence-based, patient-centered care. While many studies recognize that earlier correction can improve psychosocial outcomes, future research can better elucidate the optimal age for repair. In clinics, surgeons can implement standardized psychosocial screening with age-appropriate and deformity-specific assessment tools to understand their patients’ psychological risk. The integration of psychologists within multidisciplinary chest wall clinics can further support those at risk and provide pre-operative counseling, coping strategies, and post-operative support.

## 9. Conclusions

Chest wall deformities impose a significant psychosocial burden on adolescents and young adults, often disproportionate to their physiologic impairments. Perceived chest appearance, rather than anatomic severity, is the strongest predictor of psychological risk. While patients’ anxiety and depression do not always meet formal diagnostic thresholds, they have substantial impacts on daily functioning and QoL. Treatment of PE and PC through surgical and non-surgical approaches has positive effects on body image, self-confidence, and overall QoL and has led surgeons to advocate for these domains to be considered core measures in chest wall evaluations. Early psychosocial evaluation and psychological support are important components of a holistic care model aimed at mitigating long-term emotional distress and optimizing patient outcomes.

## Data Availability

No new data were created or analyzed in this study. Data sharing is not applicable to this article.

## Abbreviations

The following abbreviations are used in this manuscript:

PE	Pectus Excavatum
PC	Pectus Carinatum
QoL	Quality of Life
HRQoL	Health-Related Quality of Life
HI	Haller Index
